# Whole genome sequence‐based association of cognitive decline and retinal thickness in the Japanese population

**DOI:** 10.1002/alz.093405

**Published:** 2025-01-03

**Authors:** Makiko Taira, Nobuo Fuse, Andrew J. Saykin, Fuji Nagami, Kengo Kinoshita, Masayuki Yamamoto

**Affiliations:** ^1^ Tohoku Medical Megabank Organization, Tohoku University, Sendai Japan; ^2^ Department of Neurology, Graduate School of Medicine, Tohoku University, Sendai Japan; ^3^ Tohoku University Advanced Research Center for Innovations in Next‐Generation, Sendai Japan; ^4^ Indiana University School of Medicine, Indianapolis, IN USA; ^5^ Center for Neuroimaging and Indiana Alzheimer’s Disease Research Center, Indiana University School of Medicine, Indianapolis, IN USA; ^6^ Graduate School of Medicine, Tohoku University, Sendai Japan; ^7^ Graduate School of Information Science, Tohoku University, Sendai Japan

## Abstract

**Background:**

Dementia is age‐related with a significant genetic contribution, yet genome‐wide association studies have not fully accounted for heritability. This discrepancy may in part be due to reliance on SNPs and small indels. Whole‐genome sequencing (WGS) data in the Japanese population may reveal population‐specific susceptibility loci for dementia. Retinal imaging with optical coherence tomography (OCT) is noninvasive, reproducible, and can detect thinning associated with progressive neurodegeneration. Association of population‐specific genetic susceptibility loci with retinal thinning and cognitive decline may reveal novel aspects of dementia risk and pathophysiology.

**Method:**

Among participants with WGS data from the Tohoku Medical Megabank Organization (ToMMo) Ophthalmology Study (“ToMMo Eye Study”), individuals with adequate quality data on retinal nerve fiber layer and ganglion cell layer thickness from spectral‐domain optical coherence tomography (SD‐OCT) scans were selected. Since retinal thinning also occurs in glaucoma, we performed a GWAS using age, sex, and 10 principal components as covariates using SAGE1.2 to obtain a set of genes responsible for glaucoma and confirm that the genotyping was successful. We then attempted to identify susceptibility loci for cognitive decline by using (1) the Mini‐Mental State Examination, Japanese version (MMSE‐J), (2) the Montreal Cognitive Assessment, Japanese version (MoCA‐J), and (3) the Mini‐COG^©^ (a simple screening for early detection of dementia, Japanese version) scores as associated factors, respectively. Furthermore, these validation results were also compared with those obtained from GWAS using imputation data performed on custom arrays (Japonica Array^TM^, v2 or NEO) for Japanese.

**Result:**

84 significant (p < 5.0E‐8) genome‐wide susceptibility loci (hg38) of RNFL were detected on 14K WGS‐based study (the top hit locus: Chr14, *SIX6* gene, P = 4.50E‐46). There were many genetic loci that have already been reported to be associated with glaucoma susceptibility, including the above locus. Among the results of GWAS for cognitive decline combining the three cognitive scores after normalization to z‐scores, several loci have shown significant susceptibility in both of RNFL and cognitive rating scale. Some loci suggested more than a high or moderate effect of altering protein efficacy.

**Conclusion:**

We present an initial WGS‐based genetic study of retinal thickness and cognitive decline in the Japanese population.

